# Identification of M5c regulator-medicated methylation modification patterns for prognosis and immune microenvironment in glioma

**DOI:** 10.18632/aging.205179

**Published:** 2023-11-06

**Authors:** Zhenyong Xiao, Jinwei Li, Cong Liang, Yamei Liu, Yuxiu Zhang, Yuxia Zhang, Quan Liu, Xianlei Yan

**Affiliations:** 1 Department of Neurosurgery, The Fourth Affiliated Hospital of Guangxi Medical University, Liuzhou 545000, Guangxi, China; 2 Department of Neurosurgery, West China Hospital, Sichuan University, Chengdu 610000, Sichuan, China; 3 Department of Pharmacy, The Fourth Affiliated Hospital of Guangxi Medical University, Liuzhou 545000, Guangxi, China

**Keywords:** glioma, m5C, RNA methylation, immune microenvironment, WGCNA

## Abstract

Glioma is a common intracranial tumor and is generally associated with poor prognosis. Recently, numerous studies illustrated the importance of 5-methylcytosine (m5C) RNA modification to tumorigenesis. However, the prognostic value and immune correlation of m5C in glioma remain unclear. We obtained RNA expression and clinical information from The Cancer Genome Atlas (TCGA) and The Chinese Glioma Genome Atlas (CGGA) datasets to analyze. Nonnegative matrix factorization (NMF) was used to classify patients into two subgroups and compare these patients in survival and clinicopathological characteristics. CIBERSORT and single-sample gene-set algorithm (ssGSEA) methods were used to investigate the relationship between m5C and the immune environment. The Weighted correlation network analysis (WGCNA) and univariate Cox proportional hazard model (CoxPH) were used to construct a m5C-related signature. Most of m5C RNA methylation regulators presented differential expression and prognostic values. There were obvious relationships between immune infiltration cells and m5C regulators, especially NSUN7. In the m5C-related module from WGCNA, we found SEPT3, CHI3L1, PLBD1, PHYHIPL, SAMD8, RAP1B, B3GNT5, RER1, PTPN7, SLC39A1, and MXI1 were prognostic factors for glioma, and they were used to construct the signature. The great significance of m5C-related signature in predicting the survival of patients with glioma was confirmed in the validation sets and CGGA cohort.

## INTRODUCTION

Glioma is a common intracranial tumor with constant evolution, frequent recurrence and poor prognosis [1]. The 2021 edition of the WHO Classification of Tumors of the Central Nervous System classifies gliomas into grades 1 to 4, with grades 1 and 2 being low-grade gliomas and grades 3 and 4 being high-grade gliomas [2, 3]. Subtypes defined by genomic and epigenomic alterations present distinct prognostic outcomes, such as IDH mutation and 1p19q chromosomal deletion [4]. Despite recent progress in the therapy for glioma, including chemotherapy, surgery, and radiotherapy, patients are still associated with unfavorable outcomes. Recent studies have demonstrated that the expression of molecular biomarkers is an essential factor for cancerous prognosis [5–9]. As a result, the molecular differences among various gliomas can help oncologists identify the prognostic biomarkers and therapeutic targets for glioma patients.

Epigenetic modifications which include DNA methylation, histone modification, and chromatin remodeling have been reported to play roles in the occurrence and progression of malignant tumors [10, 11]. In the past decades, with the advanced development of high-throughput sequencing technologies, the concern of scientists has been shifted to RNA modification [12]. RNA modifications are widely distributed in messenger RNA (mRNA), transfer RNA (tRNA), and long non-coding RNA (lncRNA), with over 100 types identified at present [13]. RNA modification is an approach to regulate genes at post-transcriptional level, including N1-methyladenosine (m1A), N6-methyladenosine (m6A), 5-methylcytosine (m5C) and N7-methylguanosine (N7G) [13]. Among them, m5C is a widespread mRNA modification driven by the NOL1/NOP2/sun domain and TRDMT1 in eukaryotes [14–16]. At the post-transcriptional level, m5C RNA modifications can be dynamically regulated by a series of mediator proteins known as “writers, erasers, and readers” [17]. The “writers” are RNA-methylases NSUN1-7, DNMT1, DNMT2, DNMT3A, and DNMT3B [18]. The main “eraser” is TET2, which acts as a demethylase [19]. And ALYREF is an essential “reader” protein, which can recognize and bind to m5C sites on mRNA. Previous researches have illustrated that RNA modification can dynamically regulate important biological processes, including protein synthesis, cell proliferation, cell differentiation, and even stress response [20, 21]. Furthermore, it is clearer and clearer that abnormal expression of m5C RNA methylase might assist the development of many malignant tumors. NSUN1, NSUN2, and NSUN4 have been proved to be up-regulated in various malignant tumors, including breast cancer, gallbladder cancer, bladder cancer, prostate cancer, and cervix cancer [22–25].

Tumor immune microenvironment (TIM) has also been proved to play an important role in the initiation and development of cancers, and has a dramatic effect on the prognosis of cancer patients [26–29]. Immune subtyping of tumors is important in establishing therapy strategies and assessing prognosis of patients [30]. Several studies have illustrated the correlation between TIM and RNA modification. As for gastric cancer, m6A modification can estimate the extent of tumor inflammation, TIM stromal activity and genetic variation. Low m6A scores showed inflamed TIM phenotype and strong response to anti-PD-1/L1 immunotherapy [31]. In addition, m6A modification was decreased in the high-immunity phenotype of lung cancer, suggesting that m6A might assist immune activities and provide possible strategy for immunotherapy [32]. Nevertheless, the potential functions of m5C modification in TIM are still unclear, particularly in glioma. As a result, exploration of immune infiltration characteristics regulated by m5C RNA modification may be meaningful for the immunotherapy of glioma.

Here, for the purpose of studying the potential value of m5C and novel prediction model for glioma, WGCNA, CoxPH and LASSO were performed to select candidate genes which may play roles in m5C and immune infiltration. The results were further validated by external datasets. In addition, the novel prediction model presented a high capacity for predicting patients’ prognosis. The possible prognostic biomarkers were also identified to help the clinic therapy for glioma.

## MATERIALS AND METHODS

### Dataset collection and data procession

The LGG (n=510) and GBM (n=153) datasets of TCGA were obtained from the University of California Santa Cruz (UCSC) Xena browser. The transcriptome data were RNA-seq (level 3, HTseq-FPKM data) with complete clinical information, including age, grade, IDH status, gender, survival time. From CGGA, the gene expression data and corresponding clinical information of 388 GBM and 625 LGG were downloaded as the validation cohort. Batch effects were removed by “sva” packages before analysis [33, 34]. The single-cell data set was derived from the Gene Expression Omnibus (GEO) database (GSE103224), with a total of 8 glioma patients. We carry on the quality control processing, minGene=200, maxGene=6000, pctMT=3. Manual cell types are annotated automatically through previous literature as follows: Oligodendrocyte (OLIG1, MAG, OLIG2, PLP1, MOG, MBP), Astrocyte (S100B, AQP4, GFAP), Macrophage (AIF1, CD163, CD68), Microglial (DCX, STMN2, MAP2), Endothelial cell (VWF, CD34, FLT1, CLDN5), Fibroblasts (MYLK, PDGFRB, LUM, ACTA2), T cell (CD3D, CD3E, CD8A) [35, 36].

### Identification of m5C subgroups by unsupervised clustering

A total of 13 m5C regulators were extracted from TCGA cohorts, including 11 writers, 1 reader and 1 eraser. To study the potential biology mechanism of m5C, we clustered glioma samples into 2 subgroups by “Consensus Cluster Plus” (50 iterations, resample rate of 80%) based on the expression of 13 m5C regulators [37, 38]. To verify clustering effects, the principal component analysis was performed by “PCA” R package.

### Relation analysis between m5C subgroups and immune infiltration)

The ssGSEA was used to calculate the relative enrichment score for each immune cell through an R package called “GSVA” [39, 40]. 23 immune gene sets were collected from the study of Charoentong [41], including activated B cell, activated CD8 T cell, immature B cell, T follicular helper cell, activated dendritic cell, macrophage, natural killer T cell, neutrophil and so on. The relative abundance of each infiltrating cell was indicated by the enrichment scores calculated by ssGSEA (Supplementary Table 1). The proportions of 22 tumor infiltrating immune cells in 2 subgroups were estimated by a “CIBERSORT” R package [42, 43].

### Weighted correlation network analysis

To identify m5C-related genes, WGCNA was used to establish a gene co-expression network based on TCGA datasets. M5C subgroup related modules and hub genes were identified by “WGCNA” R package [34, 44]. All samples and genes were filtered and used to establish a scale-free network by calculating the connection strength. A scale-free topology model was determined with scale-free R^2^ ranging from 0 to 1. Topological overlap Matrix was constructed from the adjacency matrix and used to form modules by dynamic tree cut. The soft-thresholding power was set as 16, and minimal module size was [39]. The relationship between modules and clinical traits were calculated by Pearson’s correlation test, and P<0.05 was significant confidence. Genes with significances value >0.3 and module membership value >0.8 were defined as hub genes to perform following analysis.

### Construction and evaluation of m5C-related signature

We performed a scoring system to quantify the m5C modification patterns in individual tumors. TCGA datasets were separated into a training cohort (n=332) and a validation cohort (n=328) by “caret” package. In the training cohort, univariate CoxPH was conducted to determine survival-related hub genes. Then, the least absolute shrinkage and selection operator (LASSO) regression model (iteration=1000) with an elastic-net penalty was performed for further screening, using a R package called “glmnet” [45]. Finally, multivariate CoxPH was performed for constructing a m5C-related signature. Based on the median of risk scores, patients were classified in to low and high-risk groups.

### RT-qPCR verification of gene expression in glioma tissues

Total RNA was extracted from tissue specimens using Animal RNA Isolation Kit (Invitrogen, Beyotime, Shanghai, China) according to the manufacturer’s instructions, and RNA was reversely transcribed into cDNA using Transcription First Strand cDNA synthesis kit (Beyotime, Shanghai, China). Quantitative real-time PCR (qRT-PCR) analyses were quantified with BeyoFast™ SYBR Green (Beyotime, Shanghai, China). The relative expression of SEPT3, CHI3L1, PLBD1, PHYHIPL, SAMD8, RAP1B, B3GNT5, RER1, PTPN7, SLC39A1 and MXI1 were calculated based on the 2-ΔΔCt method with GADPH as an internal reference. We analyzed the expression between genes in normal brain tissue and different glioma tissues.

### Single cell analysis of m5C related gene expression in glioma

Further, to verify the importance of m5C-related genes. We performed the expression of SLC39A1, SEPT3, SAMD8, RER1, PAP1B, PTPN7, PLBD1, PHYHIPL, MXI1, CHI3L1, B3GNT5 in glioma tumor microenvironment cells. The enriched cell subpopulations of m5C-related genes were also visualized by the R package “AUCell”.

### Statistical analysis

Correlation coefficients between the expression of m5C regulators and infiltrating immune cells were computed by Spearman analysis. Survival analysis was generated by the Kaplan-Meier (K-M) curve and log-rank test to identify significance of differences. Receiver operating characteristic (ROC) analyses were performed to measure the prognostic capacity of the m5C-related signature. Mann-Whitney and Kruskal-Wallis tests were performed to determine statistical difference between clinical information and subgroups.

### Data availability statement

TCGA (https://www.cancer.gov/about-nci/organization/ccg/research/structural-genomics/tcga), CGGA (http://www.cgga.org.cn).

## RESULTS

### Landscape of variation of m5c regulation in glioma

**Figure 1:**
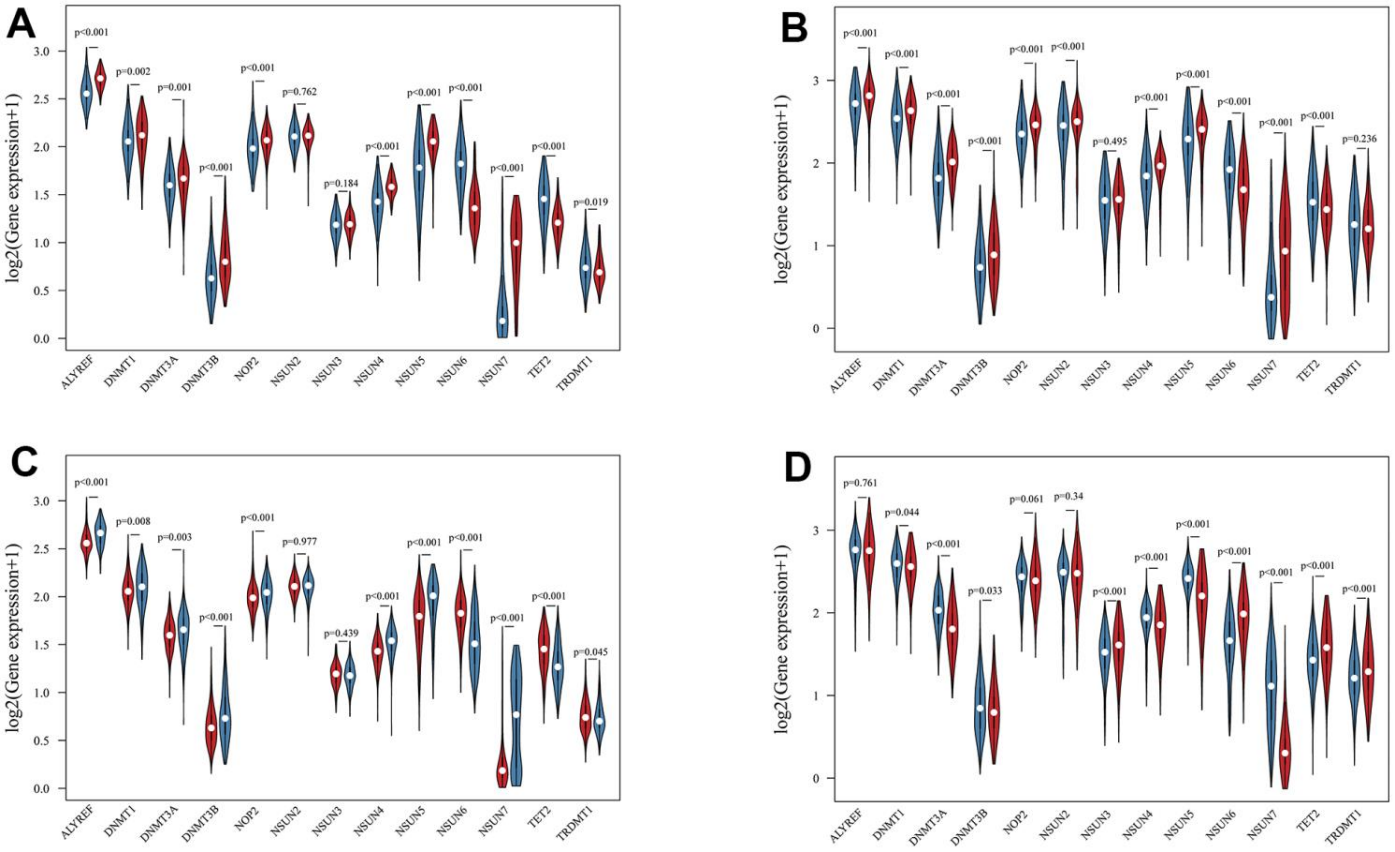
**The landscape of m5C RNA methylation regulators in glioma.** (**A**, **B**) Vioplots visualized the differentially expressed m5C regulators between LGG and GBM in TCGA (**A**) and CGGA (**B**). (blue represents LGG and red represents GBM). (**C**, **D**) Vioplots visualized the differentially expressed m5C regulators with IDH status in TCGA (**C**) and CGGA (**D**). (blue represents wild type and red represents mutation type).

**Figure 2:**
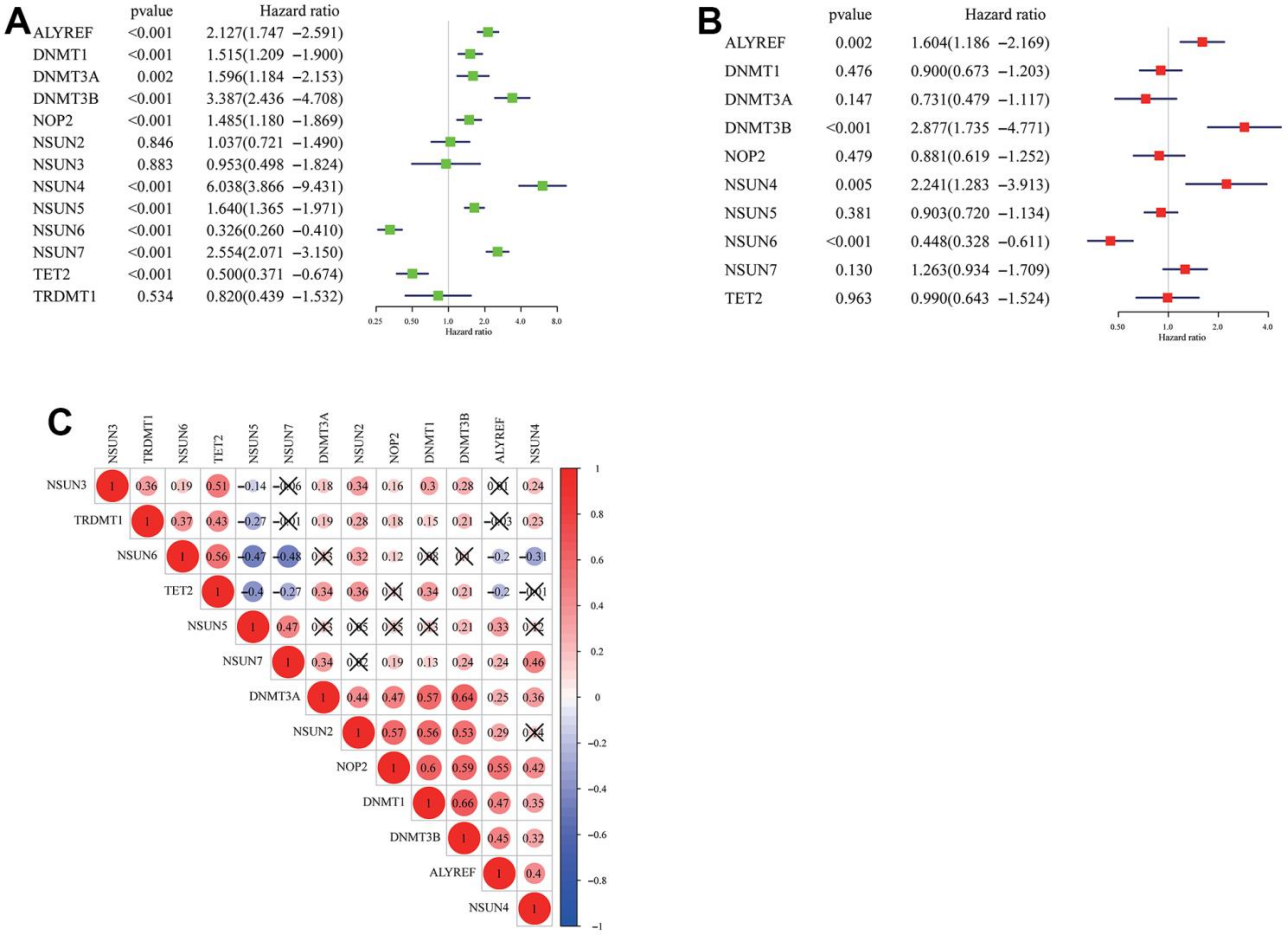
**Cox regression analysis and interaction of m5C regulators.** (**A**) Univariate CoxPH of 13 m5c regulators in the TCGA dataset. (**B**) Multivariant CoxPH of 13 m5c regulators in the TCGA dataset. (**C**) The interaction between m5c regulators in glioma.

Using transcriptome data from TCGA and CGGA datasets, we analyzed a total of 13 m5c regulators including 11 writers, a reader and an eraser in this study. Figure 1 summarized the mRNA levels of m5C regulators in glioma. Except NSUN2, NSUN3 and TRDMT1, 10 m5c regulators were differentially expressed between LGG and GBM (Figure 1A, 1B). NSUN6 and TET2 had higher expression in LGG, the others were opposite. Mutations in IDH were associated with a distinctive tumor-cell metabolism, so we analyzed the expression of m5C regulators in IDH status (Figure 1C, 1D). From the results, DNMT1, DNMT3A, DNMT3B, NSUN4, NSUN5, NSUN7 had obviously high expression, and TET2 and TRDMT1 had low expression in wild type. The prognostic value of 13 m5c regulators was revealed by univariate and multivariate Cox regression models (Figure 2A, 2B). ALYREF, DNMT3B, NSUN4, NSUN6 play a significant role in survival of glioma patients. Figure 2C presented a remarkably correlation among m5C regulators. We also analyzed the correlation among writers, readers and erasers in detail (Supplementary Figure 1). It was found that tumors with down-expressed eraser gene (TET2) had a high expression of writer or reader genes (ALYREF, NSUN5 and NSUN7). Tumors with up-expressed eraser gene (TET2) also showed high expression of writer genes (DNMT1, DNMT3A, DNMT3B, NSUN2, NSUN3, NSUN6 and TRDMT1). The above analysis displayed the pattern of expression alterations in m5C regulators, indicating that m5C regulators may play an essential role in the initiation and development of glioma.

### Identify two subgroups of gliomas by consensus clustering of m5C regulators

**Figure 3:**
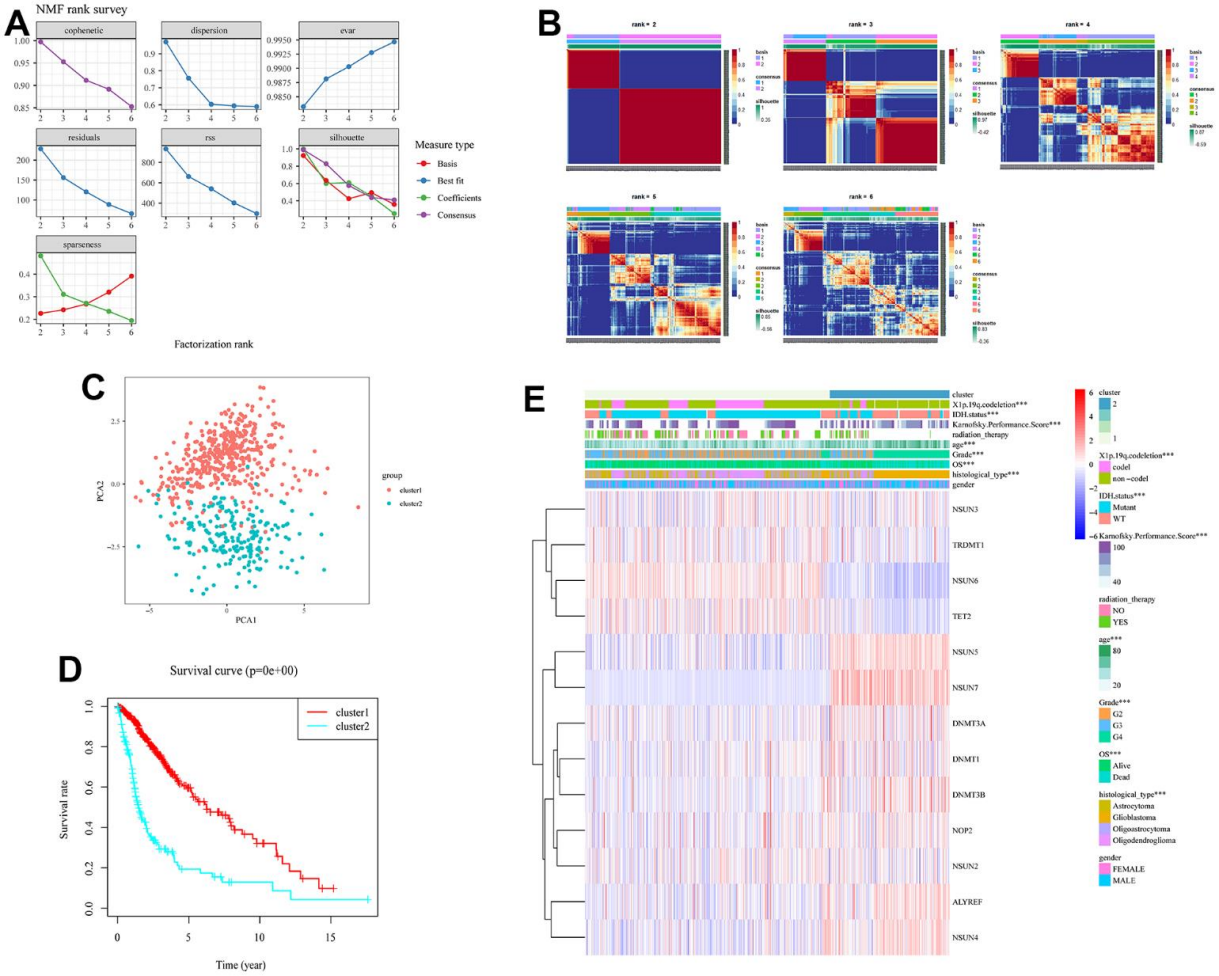
**Obtaining consensus clusters by m5c regulators.** (**A**) The relationship between cophenetic, dispersion, evar, residuals, rss and silhouette coefficients with respect to number of clusters. (**B**) The consensus map of NMF clusterin in the total TCGA cohort. Patients were clustered into subgroups based on the expression of 13 m5c regulators. (**C**) Principal component analysis for the expression profiles of 13 m5c regulators to distinguish different cluster. (**D**) The survival curve in cluster 1 and 2. (**E**) The correlation analysis of m5c regulators and clinical phenotypes in cluster 1 and 2.

To better explore the underlying biology mechanism of 13 m5c regulators, unsupervised clustering was used to cluster patients with the same characteristics. The R package of NMF was used to classify patients into 2 subgroups based on the expression of 13 m5c regulators, including 446 cases in cluster 1 and 217 cases in cluster 2 (Figure 3A, 3B). Principal component analysis proved the differences between cluster 1 and cluster 2 (Figure 3C). Cluster 1 was characterized with high expression of NSUN6, TET2, TRDMT1 and NSUN3, and cluster 2 had high expression of NOP2, DNMT1, ALYREF, DNMT3A, NSUN4, DNMT3B, NSUN5 and NSUN7 (Figure 3E and Supplementary Table 2). To further compare cluster 1 and cluster 2, we performed survival and clinical information analysis. Cluster 1 had better survival rate than cluster 2 (Figure 3D, p<0.05). As shown in the heatmap, they were significantly different in transcriptome subtype, X1p.19q.codeletion, karnofsky performance score, IDH status, age, grade and histological type (Figure 3E). Above results suggested that m5C RNA methylation is correlated with the progression and clinical phenotypes of glioma.

### The relationship between m5C regulators and immune infiltration

**Figure 4:**
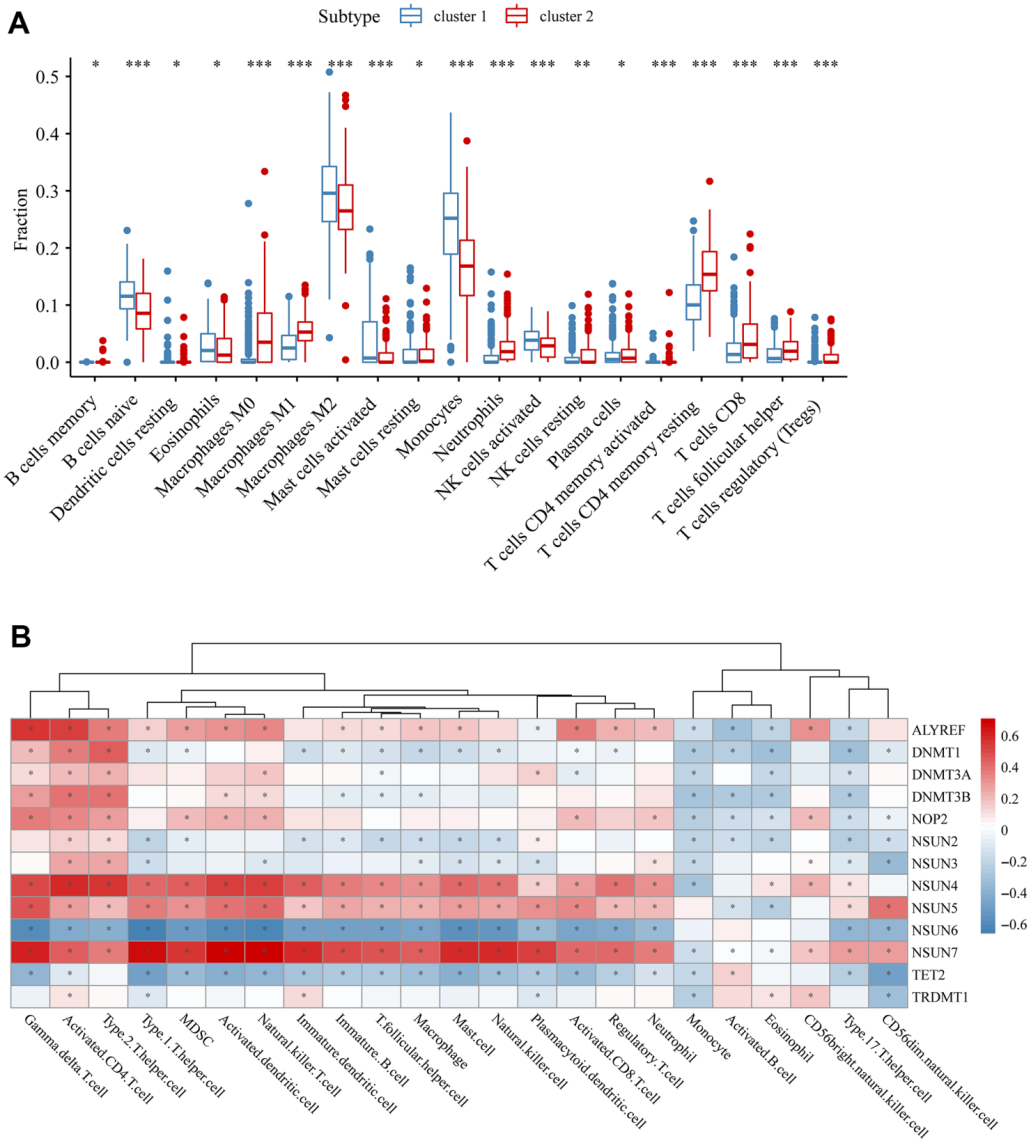
**The relationship between m5c and immune.** (**A**) The abundance of each immune cell in two clusters. The lines in the boxes were the median values. The asterisks represented the p values (*P < 0.05; **P < 0.01; ***P < 0.001). (**B**) Spearman correlation analysis of immune score and m5c genes.

Immune microenvironment has been identified as a critical regulator in prognosis and immunotherapy, so it is worth to investigate the value of m5C regulators in immunity. CIBERSORT method, a deconvolution algorithm for detecting the immune cells in tumor tissues, was used to identify the difference in infiltrating immune cells among m5C-related subgroups. We found cluster 1 was characterized by the high infiltration of B naïve cell, resting Dendritic cell, Eosinophil, Macrophage M2, activated Mast cell, Monocyte and activated NK cell. Cluster 2 had high infiltration of B memory cell, Macrophage M0, Macrophage M1, resting Mast cell, Neutrophils, resting NK cell, Plasma cell, activated CD4 memory T cell, resting CD4 memory T cell, T CD8 cell, T helper cell and T regulatory cell (Figure 4A). Then we explored the correlation between separate m5C regulators and infiltrating immune cells by ssGSEA method. The results also presented significantly different immune infiltration between clusters (Supplementary Figure 2). We found most of m5C regulators were highly correlated with immune cells (Figure 4B). Especially, NSUN7 had obviously positive correlation with Gamma delta T cell, Type 1 T helper cell, Activated dendritic cell, Natural killer T cell and Mast cell. Therefore, we could conclude that m5C methylation might impact the immune microenvironment in glioma.

### Identification of m5C-related module and hub genes by WGCNA

**Figure 5:**
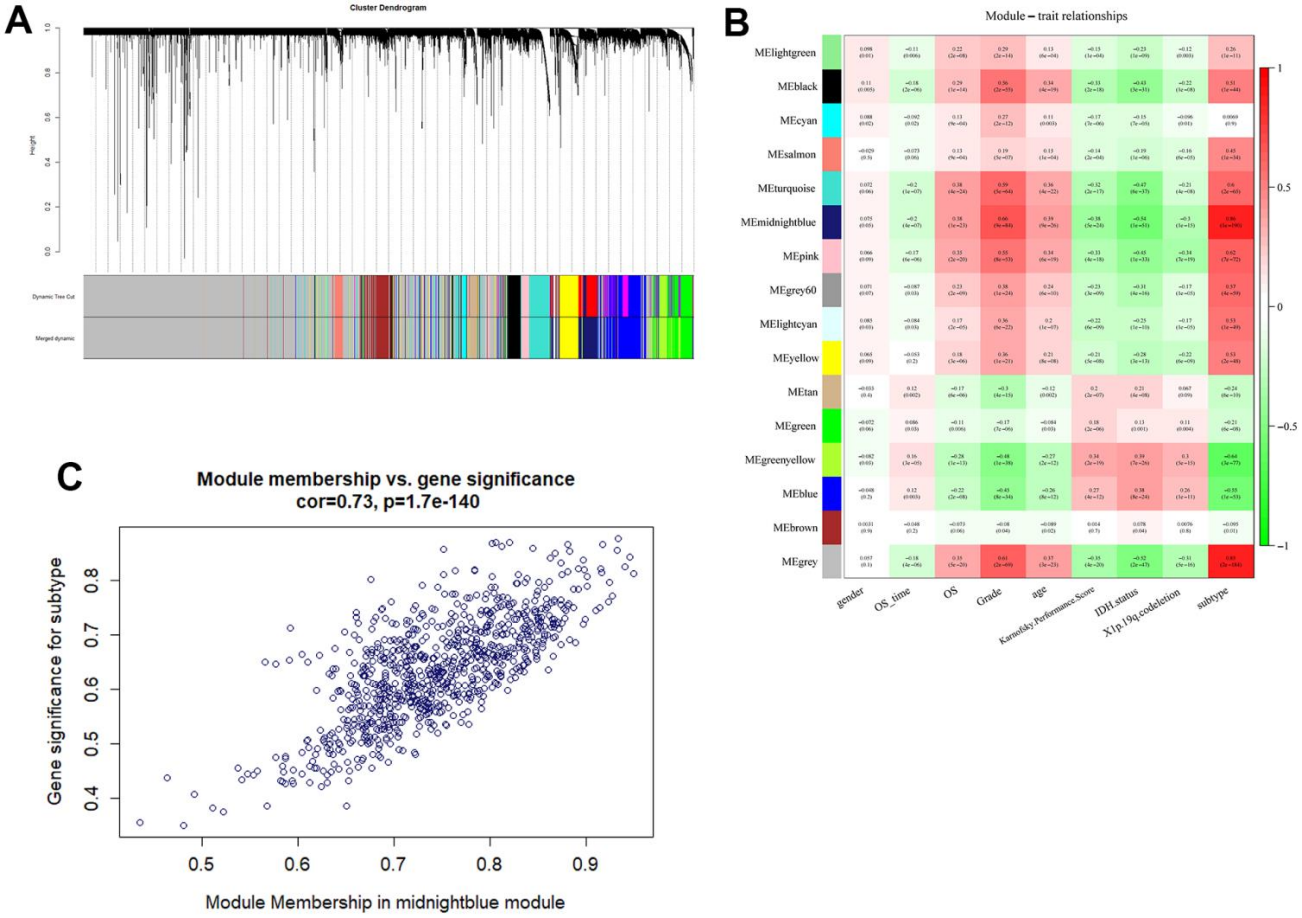
**Detection of m5c-related module by WGCNA.** (**A**) The gene was clustered based on the expression level. (**B**) Heatmap of the association between gene module and the clinical phenotype of glioma. The midnightblue module was the most correlated module of subtype characterized. (**C**) The correlation analysis between membership (MM) in and gene significance (GS) in midnightblue module.

We performed WGCNA on the TCGA datasets to investigate the hub genes that were mostly correlated with m5C methylation modification and infiltrating immune cell in glioma (Supplementary Figure 3). 16 co-expression modules were identified by setting soft-thresholding power as 16. Clinical information, including gender, survival time, survival status, grade, age, Karnofsky performance score, IDH status, X1p.19q.codeletion and m5C subtype, was included in analysis (Figure 5A, 5B). From the heatmap, the MEmidnightblue was the most correlated module of the subtype (r=0.86, P=1e-190). MEmidnightblue module contained 839 genes, and 295 genes (GS value >0.3 and MM value >0.8) were finally defined as hub genes (Supplementary Table 3).

### Generation of m5c gene signatures

**Figure 6:**
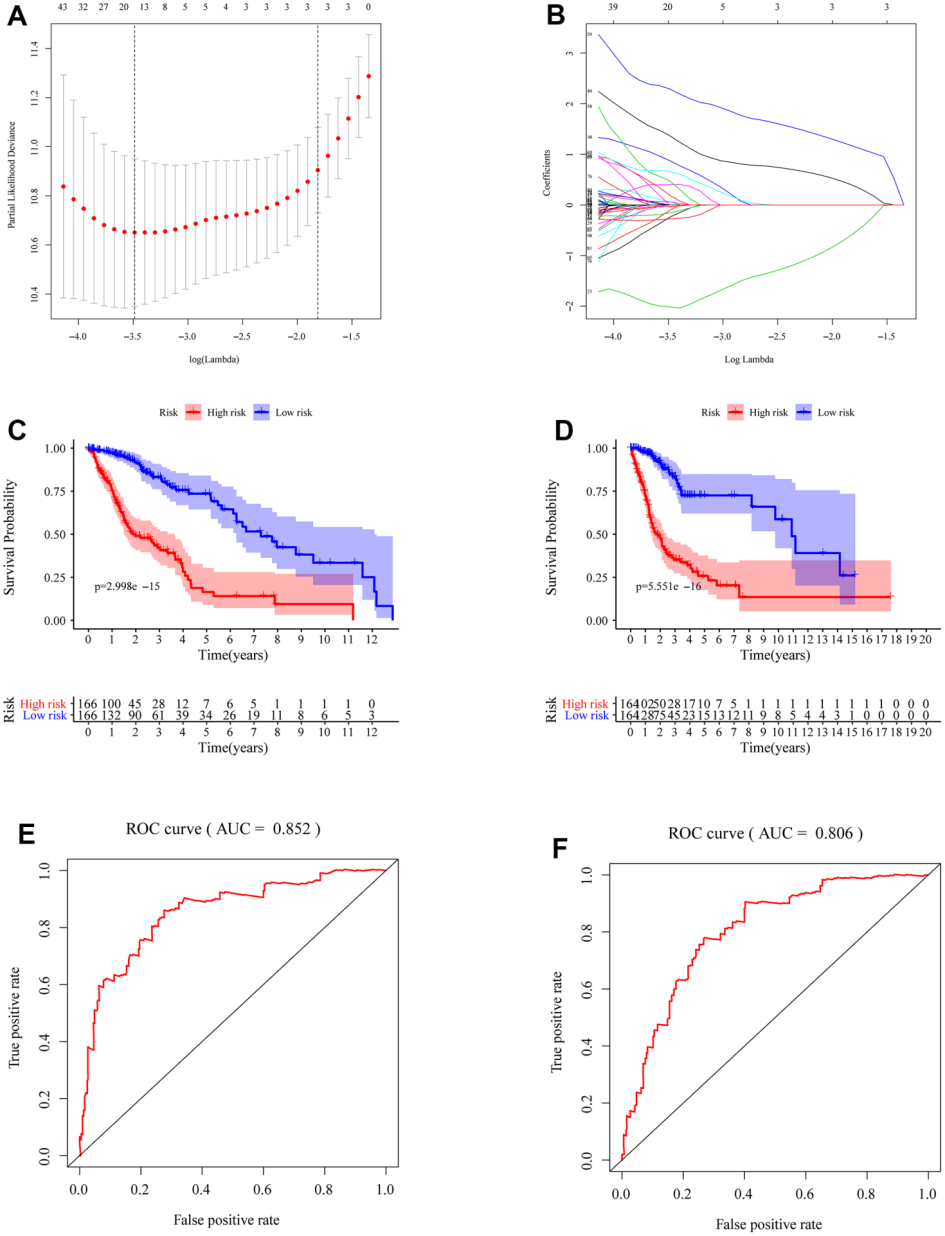
**Construction of m5c-related signature.** (**A**, **B**) Identification of prognostic genes and the coefficients constructed using the LASSO method. (**C**, **D**) Comparing survival in high- and low-risk subgroups by drawing K-M survival curves in the total TCGA cohort, training group (**C**) and validation group (**D**). (**E**) The ROC curves of patients with glioma in training group (**F**) and validation group.

**Figure 7:**
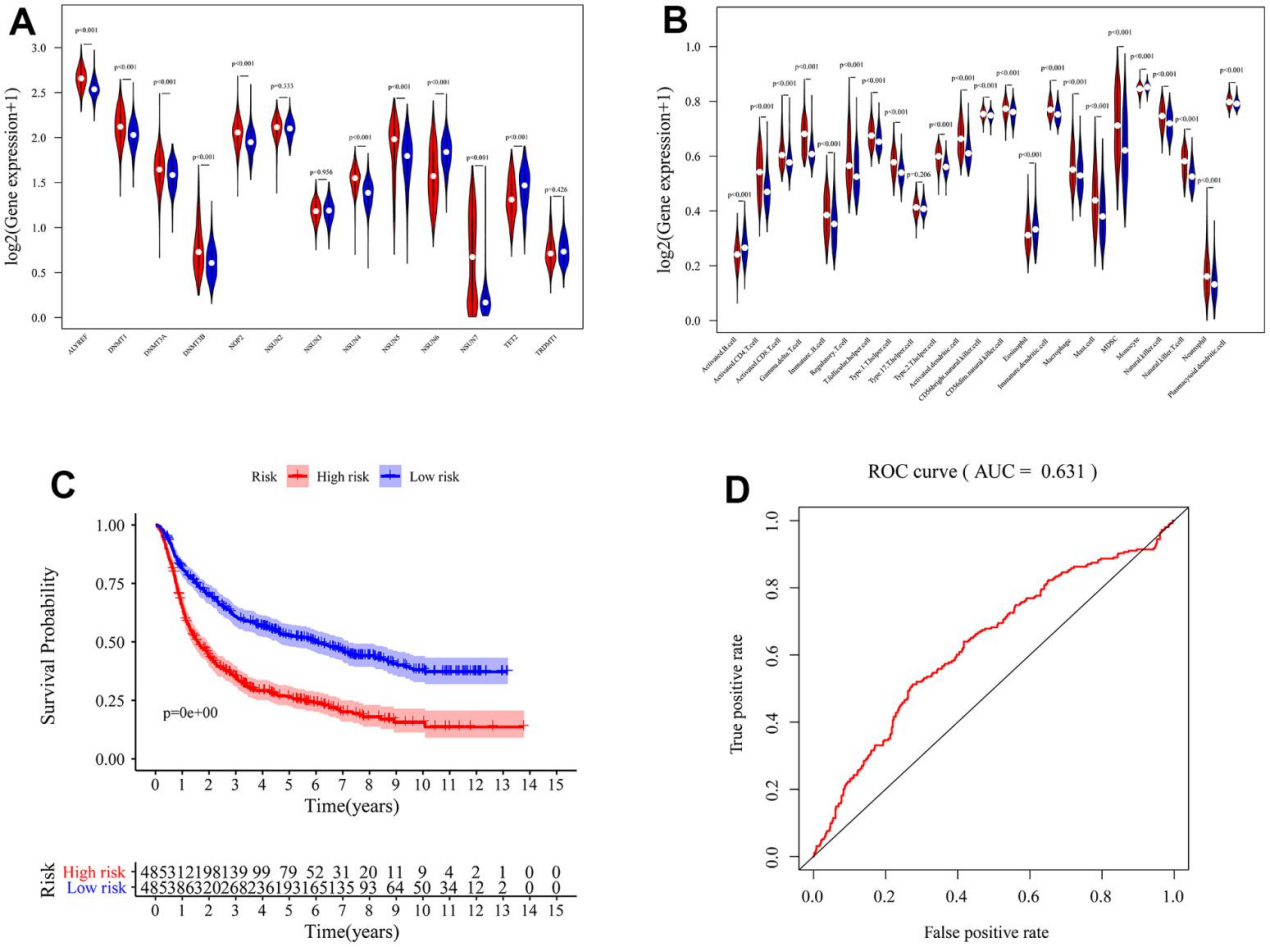
**Validation of m5c-related signature.** The differences of immune infiltration cells (**A**) and m5c expression level (**B**) between high and low risk groups. (**C**) K-M survival curves for the glioma patients of risk groups in the CGGA dataset. (**D**) The area under the curve (AUC) of ROC curves was 0.631 in predicting survival events from the CGGA dataset.

**Figure 8:**
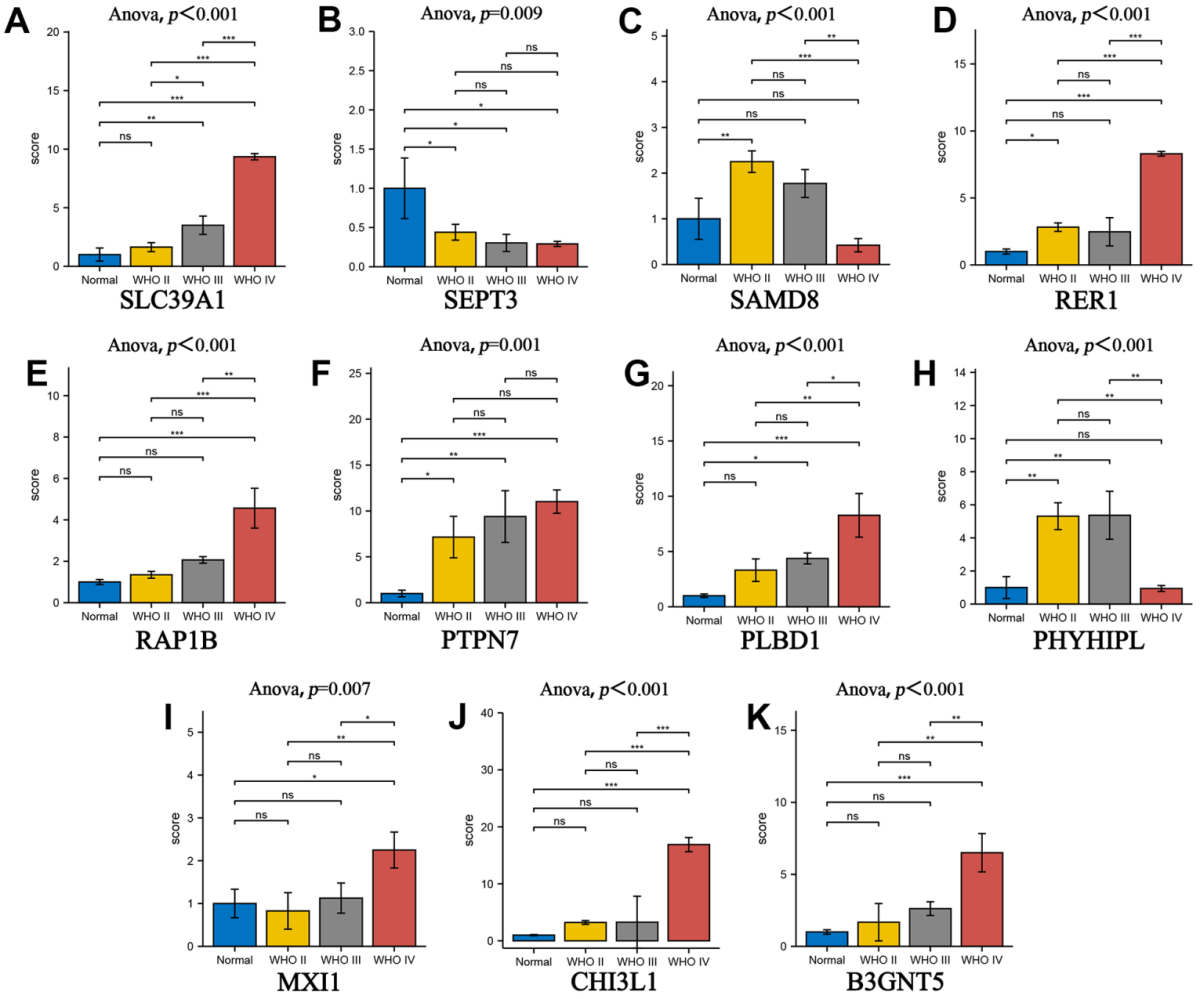
**The gene of prognostic model was used to verify the correlation of glioma grade by RT-qPCR.** (**A**–**K**) The box plot comparing the expression of key genes in different glioma grades. *P < 0.05; **P < 0.01; ***P < 0.001.

We identified the roles of m5c methylation modification in the prognosis and immune microenvironment of glioma based on the patient population. Nevertheless, the pattern of m5c methylation modification in individual patients could not be accurately predicted, so we constructed a scoring system to quantify the m5c methylation pattern. TCGA cohort was randomly divided into a training set and a validation set. Univariant CoxPH and LASSO were used to filter survival-related hub genes in the training set (Figure 6A, 6B). Finally, 11 genes constructed a m5c-related signature by multivariate CoxPH regression model (Supplementary Table 4). Based on the median risk score, patients were divided into high-risk and low-risk groups. Compared to the high-risk group, the low-risk group had obvious survival advance (Figure 6C, P=2.998e-15). Validation set also presented the same result (Figure 6D, P=5.551e-16). The ROC curve analyses in the training set (Figure 6E, AUC=0.852) and the validation set (Figure 6F, AUC=0.806) revealed promising prognosis value of the signature. From Figure 7A, 7B, there were obvious differences in m5C regulators expression and immune cell infiltration between high and low groups. We also used CGGA datasets as an external validation and results present survival advance (Figure 7C, 7D). At the same time, we conducted QRT-PCR analysis to find SEPT3, CHI3L1, PLBD1, PHYHIPL, SAMD8, RAP1B, B3GNT5, RER1, PTPN7, SLC39A1 and MXI1 have statistically significant differences in normal tissue and tumor tissue inter-tissue inter-expression groups (Figure 8A–8K). The results further confirmed that the m5C signature could predict the survival and immune environment of glioma.

### Single-cell analysis of m5c-related gene expression in glioma microenvironment

**Figure 9:**
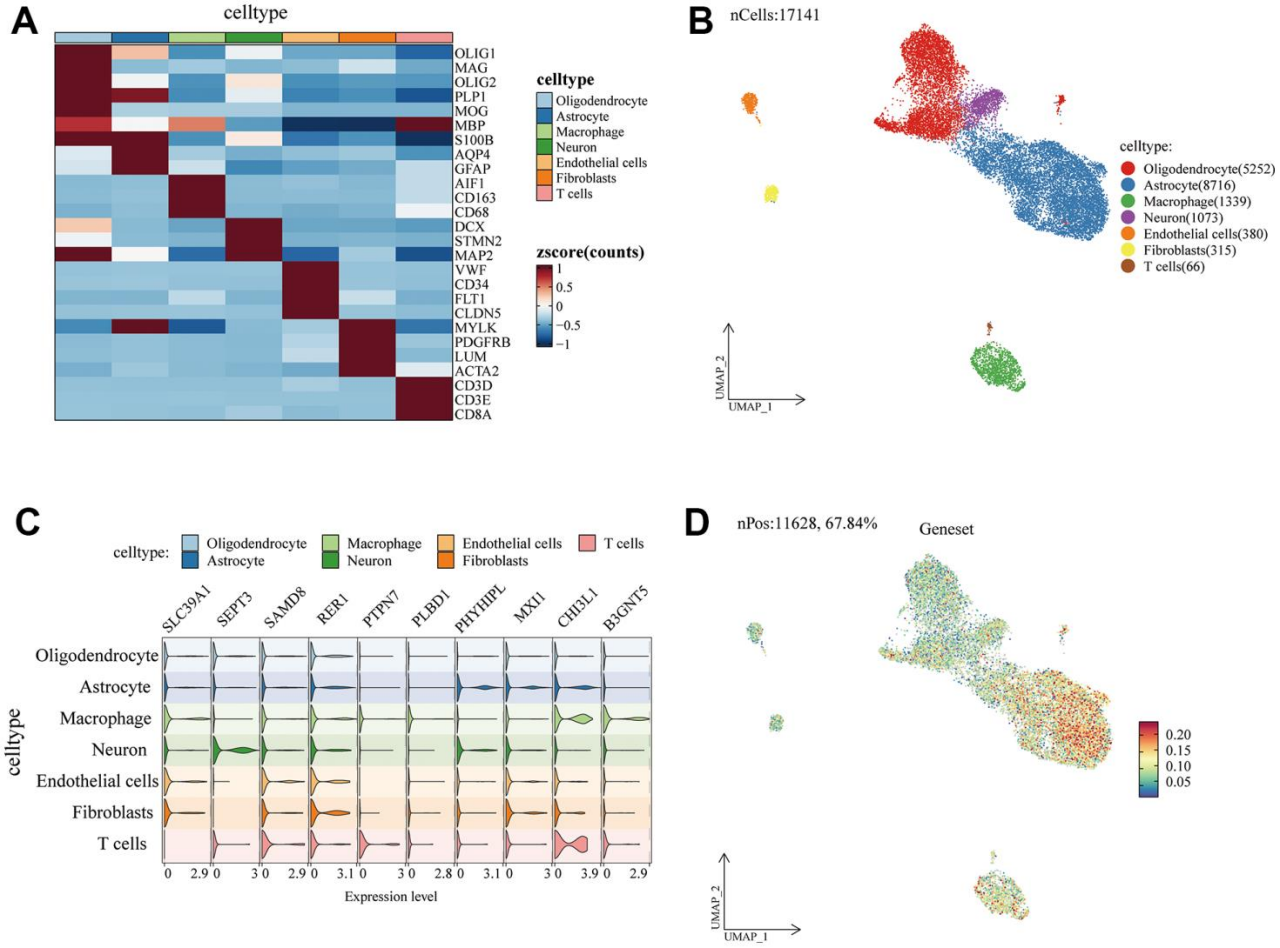
**Single-cell analysis of m5c-related gene expression in glioma microenvironment.** (**A**) Heatmap demonstrating subpopulation signature markers. (**B**) UMAP demonstrating glioma cell subpopulations. (**C**) Violin plot demonstrating m5c gene expression in cellular subpopulations. (**D**) m5c-related genes are enriched to express cellular subpopulations.

To analyze the relationship between m5c-related genes and the immune microenvironment, we performed an analysis of their gene expression in glioma monocytic cells. As shown in Figure 9A, we first performed the identification of cell subpopulation signature genes, which were categorized into a total of seven clusters of cells, namely, Oligodendrocyte, Astrocyte, Macrophage, Microglial, Endothelial cell, fibroblasts, and T cells. we found that SAMD8, RER1, MXI1, and CHI3L1 were highly expressed in most of the glioma tumor microenvironment cells (Figure 9D).

## DISCUSSION

Being regarded as the most aggressive primary tumor in the brain, glioma has become a tough task in neurosurgery [46, 47]. Aberrant RNA epigenetic modifications have been reported to play roles in the initiation and progression of cancer. For example, m6A RNA methylation regulators are important participants in the malignant progression of gliomas and are potentially helpful for prognostic prediction [48]. This indicates that epigenetic modification regulators may have a potential value in cancer diagnosis and the personalized therapy [49, 50]. Previous research only explored the effect of one m5C regulator on glioma, and did not systematically study how m5C regulator-medicated methylation modification patterns influence the progression of glioma. For instance, Wang et al. only explored the action mechanism of “writers” (NUSN1-7 genes) in glioma. However, m5C RNA modifications are not only regulated by “writers”, but also “erasers and readers”. m5C regulators can interact with each other and regulate the whole system together. Here, we studied the effect of m5c regulators on glioma, as well as the interaction among m5C regulators.

Among 13 m5C regulators, most of them were overexpressed in GBM and wild type, suggesting the important role of m5C RNA modifications in glioma. Applying univariant and multivariant CoxPH analysis, we proved that ALYREF, DNMT3B, NSUN4 and NSUN6 were independent prognostic factors for glioma. It has been proved that DNMT3A inhibits the proliferation of human glioma cells and induces cell cycle arrest [51]. At the same time, this gene ALYREF is involved in the occurrence and development of many kinds of tumors [52–54]. Knockout of ALYREF changes multiple phenotypes of liver cancer and breast cancer [53, 54]. At the same time, it is found that circRNA_104948/miR-29b-3p/MTSS1/DNMT3B pathway may be a potential candidate for targeted therapy in patients with glioma [55]. NSUN4 and NSUN6 as model genes participate in the prognostic model of renal cell carcinoma [56]. It has been reported that DNMT3B may play a critical role in the IL-6-mediated OCT4 expression and the drug sensitivity of sorafenib-resistant hepatocellular carcinoma [57]. We further explored the interaction among m5C regulators and found that an obvious association was shown among writers, erasers, and readers. For example, as an “eraser”, TET2 was negatively correlated with reader (ALYREF) and writers (DNMT3B, NSUN4 and NSUN6). These results showed that the interaction among the m5C regulators of may significantly influence the formation of different m5C modification patterns and TIM characteristics between various gliomas.

Increasing evidence showed that m6A modification may take an essential part in inflammation, innate immunity, and anti-tumor effect via the interaction among various m6 A regulators [31, 58, 59]. However, the overall TIM characteristics mediated by multiple m5C regulators have not been completely identified. Exploring the function of different m5C modification patterns in the TIM will help us to understand the anti-tumor immune response and provide a guide for more efficient immunotherapy strategy. In this study, we revealed two different m5C modification patterns. Cluster 1 was characterized by the high expression of NSUN6, TET2, TRDMT1, and NSUN3. Cluster 2 was characterized by the high expression of DNMT1, DNMT3A, DNMT3B, ALYREF, NOP2, NSUN4, NSUN5 and NSUN7. NSUN6 controls glioblastoma response to temozolomide (TMZ) through NELFB and RPS6KB2 interaction [60]. TET2 loss is associated with glioblastoma (GBM) stem cells and correlates with low survival in GBM patients [61]. Knockdown of TRDMT1 gene, may affect cancer cell fate during chemotherapy for glioma [62]. NSUN3 has been considered to be M5 C regulators in low-grade glioma [63]. The expression of DNMT1 can predict the sensitivity of gliomas to dexitabine [64]. It has been proved that DNMT3A inhibits the proliferation of human glioma cells and induces cell cycle arrest [65]. Figure 4A showed that the TIM of these two clusters were quite different. To further explore the relationship between m5C and immune activities, we used Spearman’s analysis to evaluate the correlation between m5C regulators and infiltrating immune cells. We found that m5C regulators were strongly correlated with immune cells, especially NSUN7, and the correlation of most eraser (TET2) is oppositive with other readers and writers, except NSUN6. Although there has been no research about m5cmodification and immune, the relationship between m5C and immune deserves further exploration, especially NSUN7. Recent studies focus on the activation of dendritic cells by m6A methylation. Zhang et al. found that tumor with low-expressed KIAA1429 showed more enrichment of dendritic cell infiltration, and KIAA1429-drived m6A modification can facilitate the activation of dendritic cells, so as to enhance the anti-tumor immune response [32].

Moreover, in this study, the hub genes identified by WGCNA were correlated with m5C and immune infiltration. These genes were selected as m5C-related signature genes. We used a scoring system to evaluate the m5C methylation patterns of every glioma patient—the m5C-related signature. Similar to the two clusters based on 13 m5C regulators, two groups were classified based on the median of risk score, which were also related to the expression of m5C regulators and immune cell infiltration. This illustrated that the m5C modifications were important in shaping different immune environment landscapes. To conclude, this study illustrated the regulatory mechanism of m5C modification on immune cell infiltration in glioma tissues. The m5C modification pattern may serve as a factor which can cause the difference and complexity of TIM. The full-scale evaluation of glioma m5C modification patterns can assist our study on TIM characteristics and guide more effective immunotherapy strategies.

This study has certain shortcomings and limitations. First, our study suffers from selection bias based on database analysis. Second, although the data reliability has been mutually verified from multiple databases, it still needs to be verified from more clinical experiments. Finally, more biologically relevant mechanisms need to be further verified by *in vivo* and *in vitro* experiments.

## Conclusion

The full-scale evaluation of glioma m5C modification patterns can assist our study on TIM characteristics and guiding more effective immunotherapy strategies. This study illustrated the regulatory mechanism of m5C modification on immune cell infiltration in glioma tissues. Most of m5C RNA methylation regulators presented differential expression in GBM and wild type, suggesting the important role of m5C RNA modifications in glioma. Two m5C clusters were different in survival analysis, IDH status, X1p.19q codeletion, grade, and histological type. There were obvious relationships between immune infiltration cells and m5C regulators, especially NSUN7. In the m5C-related module from WGCNA, we found SEPT3, CHI3L1, PLBD1, PHYHIPL, SAMD8, RAP1B, B3GNT5, RER1, PTPN7, SLC39A1 and MXI1 were prognostic factors for glioma, and they were used to construct a m5C-related signature. The results further confirmed that the m5C signature could predict the survival and immune environment of glioma.

## Supplementary Material

Supplementary Figures

Supplementary Table 1

Supplementary Table 2

Supplementary Table 3

Supplementary Table 4
