# Supplementary material for: Identification of M5c regulator-medicated methylation modification patterns for prognosis and immune microenvironment in glioma
Source: Aging (Albany NY). 2023 Nov 6;15(21):12275–95. doi: 10.18632/aging.205179 (PMC10683591; doi:10.18632/aging.205179)
Supplement: Supplementary Figures [file aging-15-205179-s001.pdf]

SUPPLEMENTARY FIGURES

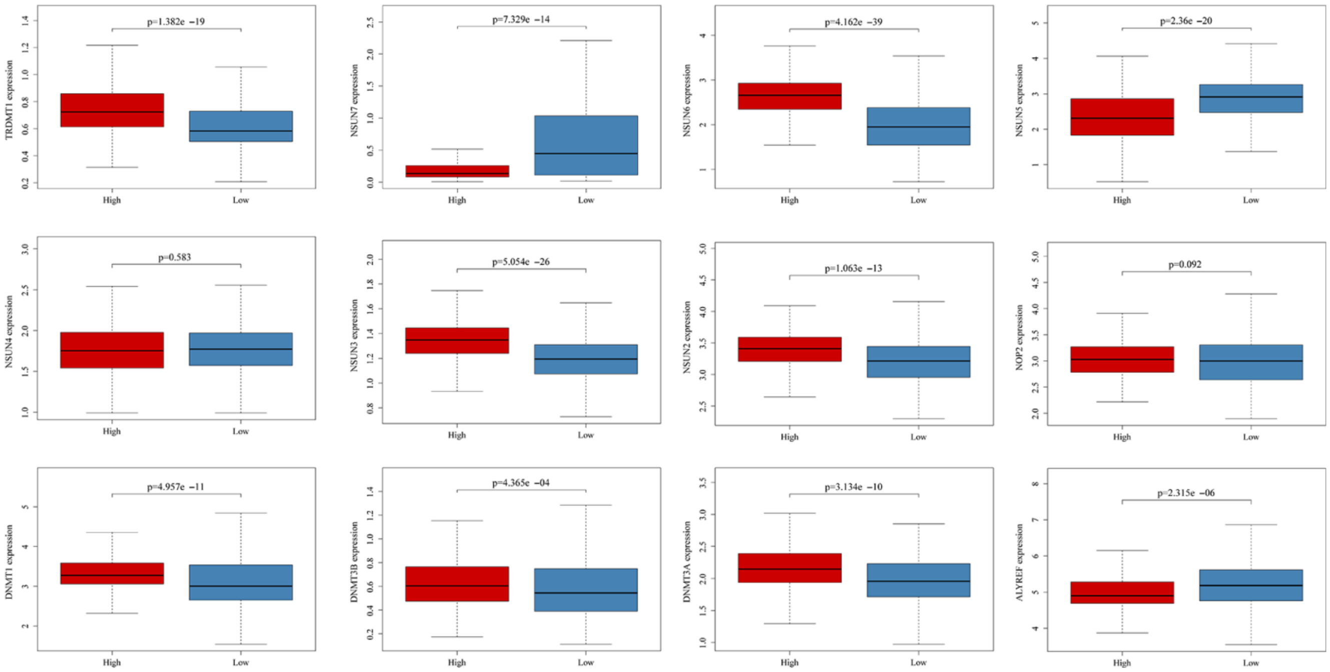

Supplementary Figure 1. Association between writer/reader gene expression and eraser gene expression.

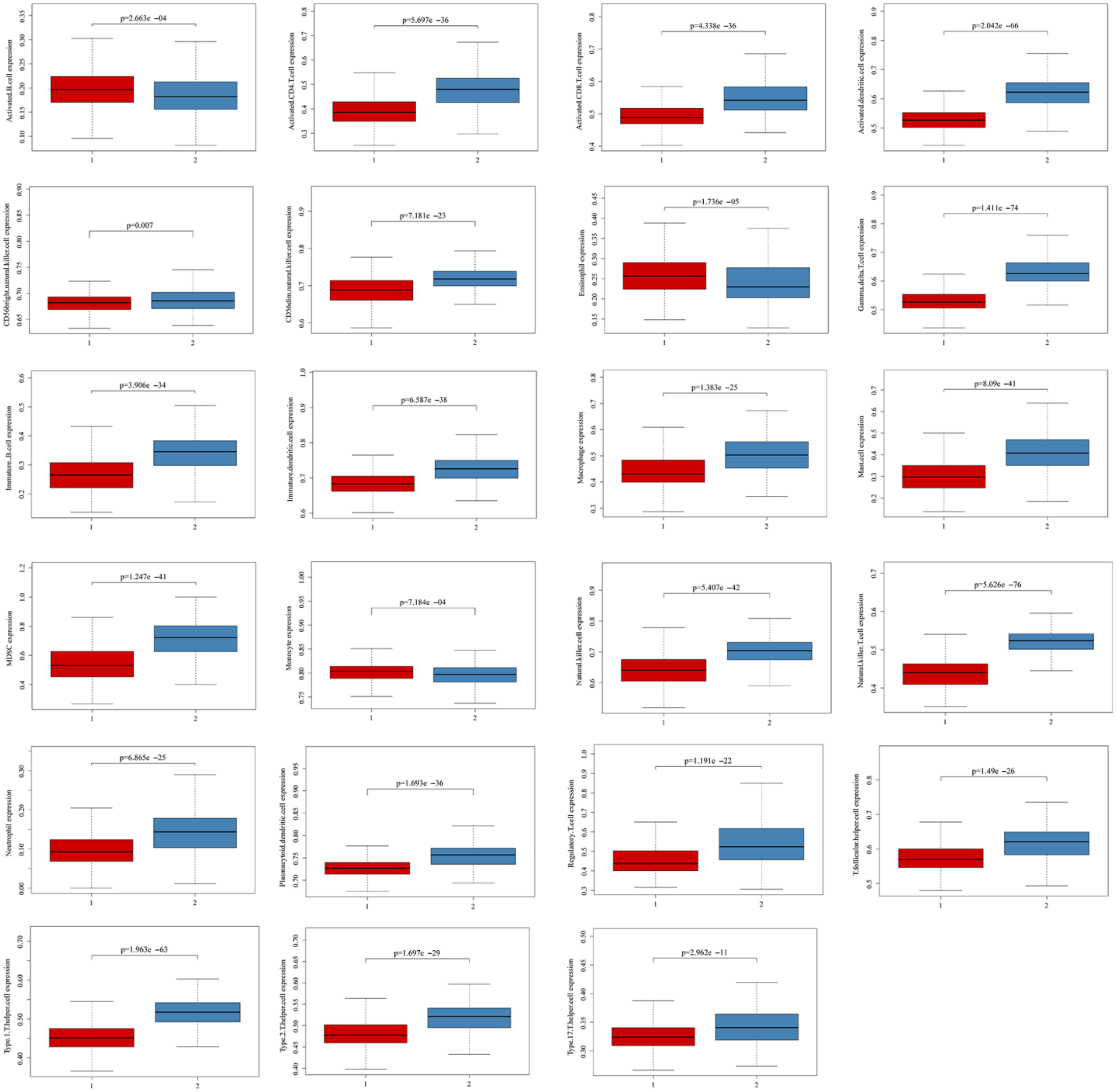

Supplementary Figure 2. The differences of separate immune infiltration cells in m5c subclusters.

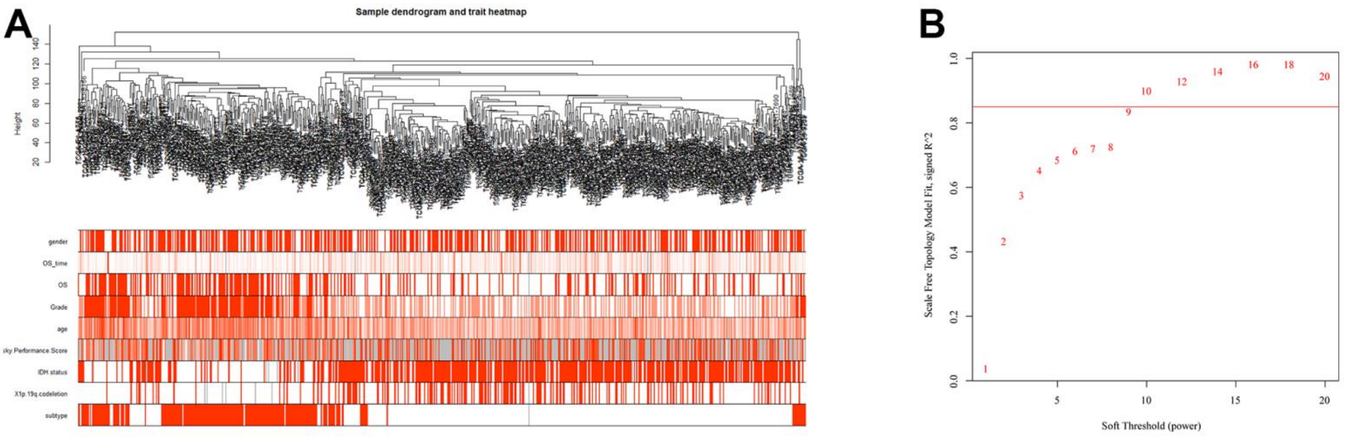

Supplementary Figure 3. The construction of a gene co-expression network. (**A**) The clusters were based on the transcriptome data from TCGA. The color intensity represented the clinical phenotypes. (**B**) Soft-thresholding power analysis was used to construct the scale-free fit index of network topology.
