# Supplementary material for: Identification of M5c regulator-medicated methylation modification patterns for prognosis and immune microenvironment in glioma
Source: Aging (Albany NY). 2023 Nov 6;15(21):12275–95. doi: 10.18632/aging.205179 (PMC10683591; doi:10.18632/aging.205179)
Supplement: Supplementary Table 2 [file aging-15-205179-s003.pdf]

Supplementary Table 2. Different expression of m5c regulators between cluster 1 and cluster 2.

Gene	Cluster 1	Cluster 2	logFC	pValue
ALYREF	4.96004073404525	5.36418000991667	0.113005682972779	6.25596851114577E-22
DNMT1	3.16902539073529	3.35876105713426	0.0838899467697152	2.62606717504477E-06
DNMT3A	2.00262148385747	2.21816515840278	0.147477025900186	1.83864159236044E-10
DNMT3B	0.581909419868778	0.763365497055556	0.391579380008811	2.08978753220134E-11
NOP2	2.98325979971719	3.13413814140278	0.071179148489837	2.68967655732733E-07
NSUN2	3.3024012305362	3.30018957917593	-0.000966511074496745	0.740780001003523
NSUN3	1.28730601264027	1.24975016685185	-0.0427153257210418	0.0255068002429809
NSUN4	1.67458119171493	1.9381427359537	0.210874496503357	6.34353266505292E-28
NSUN5	2.27724096185068	3.17409140560648	0.479056719110833	3.25938777353229E-56
NSUN6	2.62133585067421	1.69061105527778	-0.632757417257666	1.1719942893831E-71
NSUN7	0.145503581262443	1.07336134429167	2.88300926981398	8.29888406740677E-93
TET2	1.75695673890724	1.38730541231019	-0.340793239830725	1.29876107820001E-27
TRDMT1	0.71347017771267	0.640230261712963	-0.156262257610572	5.10987663027065E-07
