# Supplementary material for: Identification of M5c regulator-medicated methylation modification patterns for prognosis and immune microenvironment in glioma
Source: Aging (Albany NY). 2023 Nov 6;15(21):12275–95. doi: 10.18632/aging.205179 (PMC10683591; doi:10.18632/aging.205179)
Supplement: Supplementary Table 4 [file aging-15-205179-s005.pdf]

Supplementary Table 4. The multivariate Cox coefficients of m5c related signature.

id	coef	HR	HR.95L	HR.95H	pvalue
SEPT3	0.482554120147747	1.62020732612917	1.24661623593266	2.10575773359689	0.000308316082083268
CHI3L1	0.152505924035992	1.16474936209132	1.03059345896122	1.31636880158307	0.0145810851231528
PLBD1	-0.654897729976483	0.51949518865556	0.332151476543233	0.812506552266219	0.00410700239776913
PHYHIPL	0.369448460399882	1.44693635184989	1.04055660172608	2.01202395221149	0.0280701290239393
SAMD8	-0.686252231421828	0.503459387012789	0.289174215128679	0.876535116585351	0.0152756488909879
RAP1B	1.31919027638595	3.74039146743468	1.99591622851577	7.00957692000028	0.0000384549796866354
B3GNT5	-0.435821669489078	0.646733047887283	0.40645557723035	1.0290512879161	0.0658978547378556
RER1	1.32864693349636	3.77593084399798	1.68076512666403	8.48283529475284	0.00129389525860761
PTPN7	-0.542411766298823	0.581344493228143	0.291471087299552	1.15950238130946	0.12360012303279
SLC39A1	0.716870434802349	2.04801377822253	1.09302051566573	3.83740321034563	0.0252477509172979
MXI1	-0.507759719971774	0.60184236509044	0.363043146687824	0.997716761002842	0.0489729242836353
